# Ethiopian orthodox fasting is associated with weight reduction and body composition changes among healthy adults: a prospective cohort study

**DOI:** 10.1038/s41598-023-35060-4

**Published:** 2023-05-17

**Authors:** Alemayehu Michael, Kaleab Baye

**Affiliations:** 1 Center for Food Science and Nutrition, College of Natural and Computational Sciences, Addis Ababa University, P. O. Box: 1176, Addis Ababa, Ethiopia; 2 College of Natural and Computational Science, Hawassa University, P. O. Box: 1560, Hawassa, Sidama Regional State Ethiopia

**Keywords:** Biochemistry, Diseases

## Abstract

The Ethiopian Orthodox Christian (EOC) fasts, although adopted for religious purposes, combines aspects of energy restriction, time-restricted feeding, and a vegan dietary pattern, all of which have been independently associated with weight loss and healthier body composition. However, combined effect of these practices as part of EOC fast remains unknown. This longitudinal study design evaluated the effect of EOC fasting on body weight and body composition. Information on socio-demographic characteristics, physical activity level, and fasting regimen followed was captured through an interviewer-administered questionnaire. Weight and body composition measurements were taken before and at the end of major fasting seasons. Body composition parameters were measured by bioelectrical impedance (BIA), Tanita® BC-418®, Japan). Significant changes in body weight and body composition were observed for both fasts. Significant decreases in body weight (14/44 day fast: − 0.45; *P* = 0.004/− 0.65; *P* = 0.004), FFM (− 0.82; *P* = 0.002/− 0.41; *P* < 0.0001), and trunk fat mass (− 0.68; *P* < 0.0001/− 0.82; *P* < 0.0001) were observed after adjusting for covariates including age, sex, and physical activity. The EOC fasting regimen leads to significant reductions of body weight and compositions. Longer fasting duration led to much higher effects in body weight and body composition and may be non-pharmacological strategy in prevention or treatment of chronic diseases.

## Introduction

Over the last decades, overweight and obesity has been increasing globally and has now reached pandemic levels, spurring non-communicable diseases (NCDs)^1^. NCDs including cardiovascular diseases, cancers, and diabetes are now accounting for > 70% of premature death^2^. Unhealthy dietary patterns, compounded by low physical activity, have been identified as the major drivers of overweight/obesity and related NCDs^3^. Consequently, lifestyle interventions aiming to change dietary patterns through reduction of caloric intake have been the first line of therapies, but such approaches are challenged by the increasingly obesogenic food environment and the ubiquitous distribution of unhealthy energy-dense, nutrient-poor foods^4^.

Religious fasting has been practiced by Muslims (Ramadan) and Christians around the world for thousands of years. Recent studies have related such fasting practices to weight loss and health benefits^5^. Fasting can take many forms, including energy restriction, intermittent fasting (e.g., alternate day fasting), time-restricted feeding, and can also be accompanied by abstinence from animal source foods^6^. The length of fasting and periodicity can also vary from few days to months. Recent studies have shown that intermittent or alternate day fasting, energy restriction and vegan diet are all independently associated with weight loss and improved health outcomes^7,8^.

Devout Ethiopian Orthodox Christians fast on Wednesday and Friday, but also for an extended period during the Lent (55 days), Christmas (40 days) and Assumption (15 days) fasts^9^. The Ethiopian Orthodox fasting combines’ energy restriction and time restricted feeding through the skipping of meals (breakfast) and the adoption of vegan diet. The Wednesday and Fridays fasting also resemble a form of intermittent fasting recently named as 5:2 which symbolizes 2 days of fasting out of the seven days in a week. Altogether, devout Ethiopian Orthodox Christians fast for more than 200 days of the year. Such periodic and long-term fasting that combines different forms of fasting with a simultaneous adoption of vegan diet can have beneficial and more sustainable nutritional and health benefits. Unfortunately, long-term evaluation of this practice is scant. Natural experiment among fasting population like among the Greek Orthodox Christian have been conducted, but often targeted monks; hence, limiting the generalizability of the findings to free living adult population^10^. More recently, Sinaga et al.^11^, studied the metabolic effect of fasting among Ethiopian Orthodox followers but the study suffered inherent limitations related to retrospective cohort study design.

According to most recent estimates, more than 40% of Ethiopians are Orthodox Christians of which a large majority practicing periodic fasting. Therefore, the present study aimed to evaluate the effect of Ethiopian Orthodox Christian fasting on body weight and body composition by comparing outcomes among fasters that fasted the assumption, nativity and Wednesday and Friday fasts with non-fasters.

## Methods and design

### Study subjects

A total of 140 subjects (70 fasting and 70-non-fasting) Orthodox Christian followers residing in Hawassa city administration and meeting the inclusion criteria were approached. The inclusion criteria were as follows: apparently healthy subjects from Orthodox Christian communities within the age range of 30–45 years, permanently residing in the area, and not smoking or drinking alcohol (1 or 2 serving/week). Subjects were permanently living in the study area and had no plans of leaving before the completion of the study. The exclusion criteria were as follows: subjects with any health abnormality, pregnant or lactating women, and those planning to leave the study site before the completion of the study.

### Ethical consideration

The protocol and the questionnaires were reviewed and cleared by the ethical review board of Natural and Computational Science of Addis Ababa University, Ethiopia (IRB/035/2018). All methods were performed in accordance to the Helsinki Declaration ethical principles for medical research involving human subjects. The nature of the study was fully explained to the study participant and written informed consent was obtained from each participant prior the study. The collected data were kept confidential.

### Study design, sampling, and sample size

**Figure 1:**
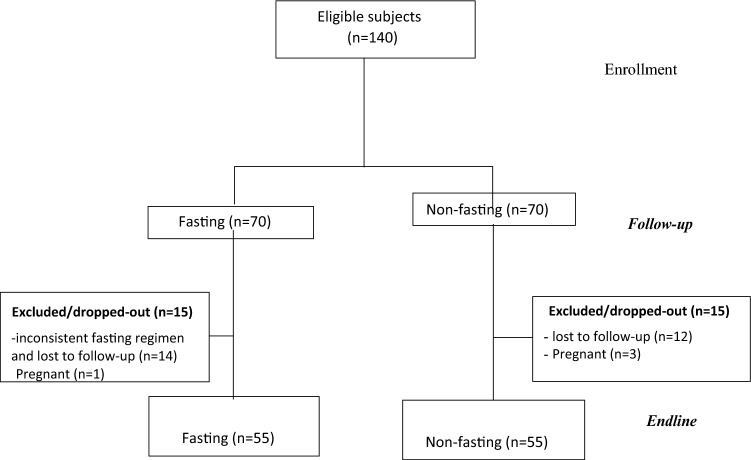
Flow diagram of study design and participant allocation throughout the study.

This study was conducted during the Assumption and Christmas fasting periods from August 2018 to January, 2019 in Hawassa, Ethiopia. The sample size was calculated using GPower to allow comparison between two groups, assuming α = 0.05, power = 0.85, a medium effect size (µ1-µ2)/α = 0.5, correlation of repeated measures (ρ) = 0.6 and considering repeated measures of four time points, as follows:

1 $$N = \frac{{2\left( {z_{\alpha } + z_{\beta } } \right)^{2} \left( {1 + \left( {n - 1} \right)\rho } \right)}}{{n\left[ {\left( {\mu_{1} - \mu_{2} } \right)/\sigma } \right]}}$$

where σ^2^ is the assumed common variance in the two groups, µ_1_ − µ_2_ is the difference in means of the two groups; n is the number of time points and ρ is the assumed correlation of the repeated measures. This yielded a minimum sample size of 38 subjects per group, which was augmented to 55 subjects per arm after accounting for a maximum of 30% loss to follow-up. A total of 110 subjects (55 per arm) completed the study (Fig. 1).

### Fasting regimen

The fasting group strictly followed the Ethiopian Orthodox fasting practice by skipping at least one meal (breakfast), then strictly adhering to a vegan diet devoid of all forms of animal source foods. The fasting group fasted the: (i) assumption fast (15 days), and (ii) Nativity/Christmas fast (44 days), and (iii) Wednesdays and Friday fasts performed throughout the year except in the one month following Easter*.* In contrast, the non-fasting group continued with their normal dietary habits.

### Socio-demographic and lifestyle factors

Standardized questionnaire was used to collect data on lifestyle and demographic factors. Physical activity levels were defined as physically active if participants exercised three or more times/week for > 30 min, moderately active if exercising 2 times/week, less physically active if exercising 1times/week, and sedentary if not physically active.

### Outcome measures

Anthropometric and body composition measurements were taken at four-time points: one-week before the start and a day before the end of the Assumption fast. For the Nativity (Christmas) fast, measurements were taken a week before the start and at the end of the fast. All the repeated measurements were obtained at the same time of day (± 1 h).

### Anthropometry

Height was measured barefoot to the nearest 0.1 cm with a stadiometer. Body weight was measured twice wearing light clothing, to the nearest 100 g using digital scale. Both height and weight were measured following standard procedures. A digital electronic adult scale (ASTO) that measure both weight and height was used for measurement. Body mass index (BMI) was calculated as weight in kilogram divided by the square of height measured in meter.

### Body composition

Body composition of the subjects was measured using bio-electric impedance analyzer (Model Tanita® BC-418®, Tokyo, Japan) with a range of 0–200 kg and with an accuracy of ~ 100 g.

### Data quality control

Data collection was conducted by experienced interviewers. Anthropometric and body composition measurements were conducted by an experienced clinical technician following standard procedures.

### Statistical analysis

All data were checked for consistency and completeness, coded and entered into Statistical Package for Social Sciences (SPSS) version 23.0 software for analyses. Normality of the continuous data distribution was checked using Shapiro–Wilk test. Descriptive statistics like frequency and percentages were used to describe dependent variable in relation to different categorical data, while mean (percent increase) and SD or standard error was used for continuous data. In addition, estimate of variances in means of study groups was assessed using student’s t- test at 95% confidence interval (CI) level. A two-way ANOVA was performed to evaluate time-fast interactions. *P*-values < 0.05 were considered statistically significant.

## Results

The baseline characteristics of the subjects in the fasting and non-fasting group was comparable, with no significant difference in socio-demographic status (Table 1). Subjects in both groups were Orthodox Christians, were mostly government employees, and were in their early thirties. The mean BMI was in the normal range (< 25 kg/m^2^). The physical activity level varied between subjects, but were not significantly different between the fasting and non-fasting group. Most of the subjects had a low to moderate physical activity level.

**Table 1:** Characteristics of study subjects.

Variable	Fasting (n = 55)	Non-fasting (n = 55)	*P*-value
Gender			0.14
Male	31(28%)	35 (32%)	
Female	24 (22%)	20 (18%)	
Age (Mean ± SD)	30.15 ± 5.54	31.27 ± 5.07	
Married (%)	57%	58%	0.68
Monthly ^§^income (mean ± SD)	3641.81 ± 1579.77	3790.73 ± 1647.76	0.423
Education level			0.53
Completed high school	5	6	–
Undergraduate level training	40	42	
Graduate level training	10	7	
Religion	Orthodox Christian	Orthodox Christian	
Employment (%)			–
Government job	40	45	
Business/Sale	15	10	
Physical activity level			
Sedentary	5	5	–
Low	31	32	
Medium	16	15	
High	3	3	

*P*-values are from independent t-tests, *SD* standard deviation.

At baseline, subjects in the fasting and non-fasting group had comparable body composition parameters except for trunk fat mass that was significantly higher in the non-fasting group (Tables 2 and 3). For 14 days fasting, significant decreases in weight, FFM, and trunk FFM were observed when fasting groups compared to non-fasting group (*P* < 0.05). However, significant increase in fat percent, fat mass and trunk fat mas were observed in the fasting compared to non-fasting group (*P* < 0.05). Slight, but non-significant decrease in BMI was found for 14 days fast (Table 2).

**Table 2:** Body composition differences between fasting and non-fasting subjects before (baseline) and after (endline) 14 days fast.

Variables	Baseline				Endline (After 14 days fast)			
	Fasting (n = 55)	Non-fasting (n = 55)	Difference [95%CI]	*P*-value	Fasting (n = 55)	Non-fasting (n = 55)	Difference [95%CI]	*P*-value
	Mean ± SD				Mean ± SD			
Weight (Kg)	62.9 ± 9.3	65.6 ± 7.7	− 2.6 [− 5.7, 0.6]	0.116	62.5 ± 9.4	66.3 ± 7.4	− 3.8 [− 7, − 0.6]	0.019
BMI (kg/m^2^)	23.4 ± 2.7	23.9 ± 2.1	− 0.4 [− 1.3, 0.5]	0.386	23.2 ± 2.7	23.9 ± 2.1	− 0.7 [− 1.6, − 0.2]	0.107
Fat percent	28.6 ± 5.7	27.3 ± 6.3	1.3 [− 0.9, 3.6]	0.250	30.5 ± 9.2	27.3 ± 6.6	3.1 [0.1, 6.1]	0.043
Fat mass	18.1 ± 4.7	18.6 ± 5.6	− 0.4 [− 2.4, 1.5]	0.653	19.6 ± 4.6	17.6 ± 5.6	1.9 [0.04, 3.9]	0.045
FFM	44.9 ± 6.9	47.0 ± 6.7	− 2.1 [− 4.7, 0.4]	0.104	43.2 ± 7.2	48.8 ± 6.7	− 5.6 [− 8.2, − 3.0]	< 0.001
VFR	4.5 ± 2.6	4.3 ± 2.8	0.1 [− 0.8, 1.2]	0.657	4.5 ± 2.6	4.3 ± 2.8	0.1 [− 0.9, 1.3]	0.834
T. fat percent	23.4 ± 7.6	23.4 ± 8.9	− 0.01 [− 3.2, 3.1]	0.995	23.7 ± 7.5	23.6 ± 8.7	0.1 [− 2.9, 3.2]	0.941
T. fat mass	7.6 ± 2.2	8.8 ± 3.4	− 1.05 [− 2.1, 0.2]	0.055	7.8 ± 2.2	10.1 ± 5.1	2.5 [− 4, − 1]	0.001
T. FFM	24.8 ± 2.9	24.5 ± 2.1	− 0.2 [− 1.2, 0.7]	0.613	24.2 ± 3	23.7 ± 2.1	− 1.0 [0.1, 2]	0.037

*BMI* body mass index, *CI* confidence interval, *SD* standard deviation Fat%, fat percent, *FFM* fat free mass, *VFR* visceral fat rating, *T.fat percent* Trunk fat percent, *T.fat mass* Trunk fat mass, *T.FFM* Trunk fat free mass.

*P*-values are for two-sample t-test.

**Table 3:** Body composition differences between fasting and non-fasting subjects before (baseline) and after (endline) 44 days of fast.

Variables	Baseline				Endline (after 44 days fast)			
	Fasting (n = 55)	Non-fasting (n = 55)	Difference [95%CI]	*P*-value	Fasting (n = 55)	Non-fasting (n = 55)	Difference [95%CI]	*P*-value
	Mean ± SD				Mean ± SD			
Weight	62.9 ± 9.3	66.0 ± 7.4	− 3.1 [− 6.3, 0.04]	0.053	62.2 ± 9.2	67.3 ± 7.4	− 5.1 [− 8.2, − 1.9]	0.002
BMI	23.5 ± 2.9	23.8 ± 2.1	− 0.3 [− 1.3, 0.6]	0.491	23.1 ± 2.6	24.3 ± 2.1	− 1.2 [− 2.1, − 0.3]	0.009
Fat percent	28.8 ± 5.8	27 ± 6.3	1.8 [− 0.5, 4.0]	0.126	29.4 ± 5.9	26.9 ± 5.9	2.4 [0.2, 4.6]	0.036
Fat mass	18.1 ± 4.6	18.7 ± 5.8	− 0.3 [− 2.3, 1.7]	0.757	18.4 ± 4.5	20.1 ± 5.6	1.9 [− 3.9, − 0.03]	0.047
FFM	44.7 ± 6.9	47.2 ± 6.7	− 2.5 [− 5.1, 0.06]	0.056	44.3 ± 6.9	47 ± 6.5	− 2.7 [− 5.2, − 0.2]	0.036
VFR	4.5 ± 2.6	4.3 ± 2.8	0.1 [− 0.9, 1.2]	0.807	4.5 ± 2.6	4.3 ± 2.8	0.1 [− 0.9, 1.3]	0.807
T. fat percent	23.3 ± 7.6	22.8 ± 7.5	0.4 [− 2.4, 3.3]	0.895	23.6 ± 7.5	23.4 ± 7.6	0.2 [− 2.6, 3]	< 0.001
T. fat mass	7.5 ± 2.2	8.8 ± 3.2	− 1 [− 2.1, 0.2]	0.085	7.9 ± 2.2	10 ± 3.9	2.6 [− 3.3, − 1.1]	< 0.001
T. FFM	24.2 ± 2.9	24.1 ± 2.3	0.04 [− 0.9, 1.1]	0.932	24.1 ± 2.8	25.3 ± 2.2	− 0.2 [− 2.1, − 0.2]	0.021

*P*-values are for two-sample t-test.

*BMI* body mass index, *CI* confidence interval, *SD* standard deviation, *Fat%* fat percent, *FFM* fat free mass, *VFR* visceral fat rating, *T.fat percent* Trunk fat percent, *T.fat mass* Trunk fat mass, *T.FFM* Trunk fat free mass.

Similarly, for 44 days fasting, significant decreases in weight, FFM, and trunk FFM were observed when fasting groups compared to non-fasting group (*P* < 0.05). However, significant increase in fat percent, fat mass and trunk fat mass were observed for 44 days of fasting in the fasting compared to non-fasting group (*P* < 0.05) (Table 3).

For both 14 days and 44 days fast, insignificant change were observed in visceral fat rating (VFR) (Tables 2 and 3).

The two-way ANOVA analyses showed that there was as significant time x fasting interaction for fat percent and trunk fat mass in the 14 days fast, while this was only significant for trunk fat mass for the 44 days fasts. However, the effects of the 44-day fast were statistically significant for body weight, BMI, fat percentage, FFM, and trunk fat percentage (Table 4).

**Table 4:** The effect of fasting with time and fast-time interaction on change of body weight and body composition of healthy fasting vs non-fasting groups.

Variable		14 Days Fasting			44 Days Fasting		
		Fast	Time	Fast*Time	Fast	Time	Fast*Time
Weight (Kg)	df	1	54	1	1	44	5
	F	0.008	1.527	0.118	27.22	3.215	1.203
	*P*-value	0.928	0.063	0.733	< 0.001	< 0.001	0.319
	pη2	0.000	0.609	0.002	0.316	0.706	0.092
BMI	df	1	83	1	1	82	4
	F	0.393	1.24	1.186	5.154	3.1	1.086
	*P*-value	0.536	0.282	0.287	0.033	0.002	0.388
	pη2	0.016	0.811	0.047	0.19	0.92	0.165
Fat %	df	1	59	12	1	59	9
	F	3.485	2.653	2.158	3.069	2.399	0.326
	*P*-value	0.07	0.001	0.036	0.087	0.002	0.962
	pη2	0.086	0.809	0.412	0.071	0.78	0.068
Fat mass (Kg)	df	1	62	14	1	58	16
	F	2.918	2.374	1.7	0.706	1.125	1.133
	*P*-value	0.097	0.005	0.105	0.407	0.362	0.366
	pη2	0.084	0.821	0.427	0.02	0.657	0.347
FFM	df	1	58	20	1	55	12
	F	5.798	1.739	1.223	4.052	2.287	1.048
	*P*-value	0.022	0.051	0.302	0.052	0.003	0.426
	pη2	0.162	0.771	0.449	0.089	0.754	0.235
T. Fat percent	df	1	66	10	1	71	13
	F	0.12	0.841	0.577	1.496	3.233	4.455
	*P*-value	0.731	0.727	0.82	0.233	0.001	0.001
	pη2	0.004	0.634	0.153	0.059	0.905	0.707
T. Fat mass	df	1	65	14	1	67	11
	F	0.005	0.995	0.63	28.553	3.419	2.343
	*P*-value	0.942	0.523	0.818	< 0.001	< 0.001	0.032
	pη2	0.000	0.69	0.233	0.488	0.884	0.462
T. FFM	df	1	42	15	1	45	14
	F	5.025	18.916	2.394	0.745	0.436	0.721
	*P*-value	0.029	< 0.001	0.011	0.392	0.997	0.743
	pη2	0.09	0.94	0.413	0.015	0.297	0.174

pη2 = Partial Eta squared and *P*-values are for two-way ANOVA.

*BMI* body mass index, *Fat%* fat percent, *FFM* fat free mass, *T.fat percent* Trunk fat percent, *T.fat mass* Trunk fat mass, *T.FFM* Trunk fat free mass.

The linear regression model adjusting for age, sex, marital status, income, education level and physical activity showed that the same body composition parameters changed in the 14 and 44 days fast, but revealed that the effect size increased with increased duration of fasting (44 days vs. 14 days; Table 5).

**Table 5:** Magnitude of effect size of 14 days and 44 days of fasting on body composition.

	14 Days Fasting			44 Days Fasting		
	Cohen’s d	r_y_λ [95% CI]	*P*-value	Cohen’s d	r_y_λ [95% CI]	*P*-value
Weight (Kg)	− 0.45	0.22 [− 7.0, − 0.6]	0.004	− 0.65	0.31 [− 8.2, − 1.9]	0.004
BMI (Kg/M^2^)	− 1.624	0.15 [− 1.6,0.16]	0.110	− 0.51	0.25 [− 2.1, − 0.3]	0.09
Fat Percent (%)	0.39	0.19 [0.1,6.2]	0.160	0.41	0.19 [0.1,4.6]	0.046
Fat Mass (Kg)	0.39	0.19 [0.04, 3.9]	0.009	− 0.39	0.19 [− 3.9, − 0.03]	0.007
FFM	− 0.82	0.38 [− 8.3, − 3.01]	0.002	− 0.41	0.2 [− 5.2, − 0.18]	< 0.001
VFR	0.04	0.02 [− 0.92,1.14]	0.130	0.047	0.02 [− 0.9,1.16]	0.122
T. Fat Percent	0.014	0.007 [− 2.95,3.2]	0.001	0.025	0.012 [− 2.6,3.0]	0.004
T. Fat Mass	− 0.68	0.32 [− 3.5, − 0.9]	< 0.001	− 0.82	0.38 [− 3.7, − 1.3]	< 0.001
T. FFM	0.41	0.19 [0.06,2.0]	0.118	− 0.45	0.22 [− 2.1, − 0.17]	0.112

*BMI* body mass index, *CI* confidence interval, *Fat%* fat percent, *FFM* fat free mass, *VFR* visceral fat rating, *T.fat percent* Trunk fat percent, *T.fat mass* Trunk fat mass, *T.FFM* Trunk fat free mass.

β [95% CI] and *P*-values are from linear regression models, adjusted for age, sex, marital status, income, education level and physical activity level; Cohen's* d* = 2*t* /√(*df*); effect size (r_y_λ) = √ (*t*^*2*^* /* (*t*^2^ + *df*)).

Within the fasting group, significant changes were observed between baseline and endline on all of the body composition parameters assessed (Tables 2, 3).

## Discussion

The present study highlighted that fasting as practiced by the Ethiopian Orthodox Christians lead to significant changes in body weight and body composition. For both the 14 days (assumption) and 44 days (nativity) fasts, significant decreases in weight, FFM, and trunk fat mass were observed in the fasting than in the non-fasting group. However, the magnitude of the changes was more pronounced in the nativity (44 days) than in the assumption (14 days) fast. The decrease in fat free mass was no more significant after adjusting for sex, age, and physical activity.

The Ethiopian Orthodox Fasting can be considered as a combination of intermittent fasting, time- restricted fasting, continuous energy restriction, and adoption of a vegan diet. All these practices have been separately studied and have shown significant reductions in body weight and body fat measures^6,11^, but our study has shown that the combination of these fasting regimens also lead to significant weight loss and fat free mass loss, while also leading to a slight gain in fat during the 44 days of fast. The significant reduction in trunk fat without significant changes in visceral fat rating (VFR) in this study may suggest that fasting had more effect in reducing subcutaneous fat.

Various pathways can explain our findings. The most obvious reason is the reduced caloric intake associated with the skipping of meal and the subsequent adherence of a vegan diet that is also often lower in energy^12^. Indeed, studies evaluating energy and nutrient intake of subjects adopting orthodox fasting have shown lower energy intake, increased antioxidant and fiber intake^12,13^. The Ethiopian Orthodox fasting, with the skipping of morning meals, also led to a 16 h or more of fasting time per day. Time-restricted feeding, allowing at least 10 h of fasting time was previously found to be associated with reduced body weight and healthier body composition even in the absence of energy restriction^14^. Time-restricted feeding was reported to support robust circadian rhythms whose disruption can increase the risk of metabolic syndrome including obesity, hypertension, insulin resistance and inflammation^15,16^. Previous studies have shown that 10 h time-restricted feeding led to a reduction of − 3.3 kg body weight, − 1.09 of BMI and − 1.01% of body fat percent change in 12 weeks, but similar levels of change or more was achieved in just two weeks (assumption fast) and eight weeks (Christmas fast) with the Ethiopian orthodox fasting^14^. In addition, loss of fat free mass (FFM) was observed after adjusted for age, sex, marital status, income, education level and physical activity level in linear regression model. Although, the EOC fasts had an impact on weight and body composition, it was ineffective in modifying body weight and fat over time, probably because of compensations happening during the non-fasting period. This is explained through both “set” or a “settling” point approaches by which body weight and fat is determined and maintained^17^.

### Limitation of the study

The present study has a number of limitations that need to be considered when interpreting our findings. First, this is a natural experiment that enrolled fasting and non-fasting subjects and compared body weight and composition over time. We could not control for residual effects related to previous fasting among the fasting group, but showed that the body composition of the fasting and non-fasting group was comparable at baseline. The study would have benefited from a detailed recording of the foods consumed to characterize the differences in diet quality, which could have provided more explanations to the presence or absence of some changes.

Notwithstanding the above limitations, our study has highlighted that the fasting regimen adopted by Ethiopian Orthodox church followers can lead to significant reductions in weight, FFM, body fat and trunk fat mass. A key criticism about adopting fasting regimens for health purposes is the difficulty to adopt them by the general public.

## Conclusion

Fasting is practiced widely among Ethiopian Orthodox followers prevent the increasing burden of overweight/obesity and related non- communicable diseases. More importantly in settings where such practices are not alien and are culturally accepted, they can be practiced for health purposes in addition to religious reasons. Future studies should investigate the effect of such fasting on micronutrient status, insulin resistance, and cardio metabolic markers and further explore the effect of fasting on circadian rhythms.

## Data Availability

All relevant data are within the paper and its supporting information file.
